# Patient-targeted Googling and social media: a cross-sectional study of senior medical students

**DOI:** 10.1186/s12910-017-0230-9

**Published:** 2017-12-04

**Authors:** Aaron N. Chester, Susan E. Walthert, Stephen J. Gallagher, Lynley C. Anderson, Michael L. Stitely

**Affiliations:** 1 Otago Medical School, University of Otago, Dunedin, New Zealand; 2 Department of Women’s and Children’s Health, University of Otago, Dunedin, New Zealand; 3 Dunedin School of Medicine, University of Otago, Dunedin, New Zealand; 4 Bioethics Centre, Division of Health Sciences, University of Otago, Dunedin, New Zealand; 5 Section of Obstetrics and Gynaecology, Dunedin School of Medicine, University of Otago, Dunedin, New Zealand

**Keywords:** Patient-targeted Googling, PTG, Social media, Online professionalism, Doctor-patient relationship, Professional ethics

## Abstract

**Background:**

Social media and Internet technologies present several emerging and ill-explored issues for a modern healthcare workforce. One issue is patient-targeted Googling (PTG), which involves a healthcare professional using a social networking site (SNS) or publicly available search engine to find patient information online. The study’s aim was to address a deficit in data and knowledge regarding PTG, and to investigate medical student use of SNSs due to a close association with PTG.

**Method:**

The authors surveyed final year medical students at the Otago Medical School, University of Otago in January 2016. A subset completed focus groups that were analysed using thematic analysis to identify key themes relating to students’ attitudes towards PTG, and reasons why they might engage in PTG.

**Results:**

Fifty-four students completed the survey (response rate = 65.1%), which showed that PTG was uncommon (*n* = 9, 16.7%). Attitudes were varied and context dependent. Most participants saw problems with PTG and favoured more explicit guidance on the issue (*n* = 29, 53.7%). SNS usage was high (*n* = 51, 94.4%); participants were concerned by the content of their SNS profiles and who they were connecting with online. Participants showing high SNS use were 1.83 times more likely to have conducted PTG than lower use groups.

**Conclusions:**

The diverse attitudes uncovered in this study indicated that teaching or guidelines could be useful to healthcare professionals considering PTG. Though ethically problematic, PTG may be important to patient care and safety. The decision to conduct PTG should be made with consideration of ethical principles and the intended use of the information.

## Background

Social media and Internet use within the health sector have been rising steadily since their advent over recent decades [1, 2]. In 2013, 92% of New Zealanders were using the Internet and 80% of them held a social networking site (SNS) membership [3]. The global rise in popularity of Internet and SNS technologies is likewise seen among doctors and medical students [1, 2]. Healthcare professionals may find numerous benefits to such technologies, including easier communication and access to information. However, SNSs and the Internet also present a raft of ethical issues by blurring some boundaries of professionalism and introducing new modes of intimacy to the doctor-patient relationship. One issue is patient-targeted Googling (PTG), which involves a healthcare professional using a SNS or publicly available search engine to find patient information online [4, 5]. PTG is a relatively new phenomenon which is still in the early stages of investigation. It has been identified as an area of professional behaviour relevant to the delivery of good healthcare [1].

Since its first mention in 2010, interest in PTG has been rising with growing recognition of the impact of social networks and accessibility of personal information on professional behaviour [4]. As these technologies increase in prominence and usage in society, there may be an increased likelihood of PTG occurring. Recent studies show PTG is occurring among medical students and healthcare professionals in various countries [6–9], though no data exist for the New Zealand context. The prevalence of PTG reported in other studies is relatively low, with one Canadian survey of physicians and medical students reporting a prevalence of 14% [9]. These studies include several large surveys in countries with somewhat similar healthcare environments to that of New Zealand (Australia, Canada and the United States of America). Though there are many guidelines about use of social media for medical students, we were unable to find any guideline or policy that specifically addressed PTG. A study by Clinton et al. did provide a pragmatic framework for considering PTG, with the aim of minimising the risk of exploiting the patient, but it is unclear to what extent this framework has been adopted in explicit guidelines and policies [4]. Their study identifies problematic motivations for conducting PTG, specifically curiosity, voyeurism and habit. They also discuss how the ubiquity of SNS and the Internet makes it easier to conduct PTG without due ethical consideration [4].

Following its emergence in literature, there have been several case studies on PTG, which document both its positive and negative aspects [5, 10]. Within these case studies, it was evident that patients may believe their privacy and autonomy are being breached, which could fuel consequent distrust towards the healthcare professional. Patient distrust and resentment could strain the doctor-patient relationship, which may result in less effective care and poorer health outcomes. This is particularly relevant to the psychotherapeutic relationship, where a lack of trust may hinder any further treatment [11]. This relationship may also suffer if a healthcare professional forms a negative perception of their patient based on derogatory information found online. This information may not necessarily be false, as even accurate details about the patient could easily be misconstrued by an unwitting healthcare professional (discussed in Case Vignette 1 below) [4]. In contrast, other case studies have documented the positive potential of PTG, and its role as a crucial aspect of patient care and safety [10]. Volpe et al. describe one such case, whereby PTG revealed a patient to be deceiving doctors in order to undergo a significant and unnecessary operation [10]. If critical information is unavailable from the patient or other sources, PTG may represent a valuable and simple solution. Such instances may include unconscious patients in an emergency department or patients with advanced dementia. Within the wider literature, the care and safety of psychiatric patients was often cited as a useful role of PTG [4]. Healthcare professionals could learn more about a patient’s mental state or suicidal ideation from the content of their SNS posts, which may enable the healthcare professional to prevent harm to the patient [4]. Therefore, healthcare professionals need to balance the need for information with the ethics of PTG, whilst also considering the potential for gaining misinformation about the patient.

Online patient information exists in a variety of forms, ranging from content that patients have created about themselves (e.g. photos, videos and writings), to content created about patients by third parties (e.g. news articles and police reports). The value of this information to patient care is heavily context dependent, though conceivably, may be of greatest benefit to patients with an impaired ability to convey accurate information to the healthcare professional (e.g. emergency department and psychiatric patients). Ironically, these are also the patients in which PTG has the potential to cause the greatest harm. This harm could be caused in a variety of ways. It could result from a breakdown of the therapeutic alliance caused by the healthcare professional’s own perception of the patient (e.g. a nurse has been assaulted and does not want to treat a patient after reading online that they are a violent offender), or due to clinical decisions being unduly influenced by unreliable online information (e.g. a doctor not taking a patient’s acute abdomen seriously after reading a comment on Facebook indicating the patient is a drug seeker).

### Case vignette 1

Clinton et al. describe a case where an effective therapeutic alliance is completely eroded by PTG [4]. In this case, a psychiatrist doubted their patient’s inability to pay for their therapy sessions in full. The psychiatrist went on to search for the patient’s home address online, whereby they discovered that the patient lived in a large house in an affluent neighbourhood. Upon confronting the patient, it emerged that they were renting a room in the basement for a small fee and also performing chores around the house. The patient did not return for any further sessions [4].

### Case vignette 2

Baker et al. describe a case in which a 26-year-old woman requested a bilateral prophylactic mastectomy, without the benefit of genetic testing, due to her unsubstantiated family history of breast, ovarian, and oesophageal cancer [5]. She also claimed to have had a previous melanoma removed. The genetic counsellor searched for the patient online, in light of her inconsistent family and personal medical history. The patient was found to be soliciting donations to attend a cancer conference. She was also found to be giving newspaper interviews and blogging about her personal experience with cancer. The breast surgeon consequently declined to offer her surgery [5].

Patient safety is a ubiquitous theme among articles exploring PTG, including an article by Baker et al., who outline ten situations that may warrant PTG [5]. All of these situations have concern for patient safety at their core: “1) duty to re-contact/warn patient of possible harm, 2) evidence of doctor shopping, 3) evasive responses to logical clinical questions, 4) claims in a patient’s personal or family history that seem improbable, 5) discrepancies between a patient’s verbal history and clinical documentation, 6) levels of urgency/aggressiveness incommensurate with clinical assessment, 7) receipt of discrediting information from other reliable health professionals that calls the patient’s story into question, 8) dissonant or incongruent statements by the patient, or between a patient and their family members, 9) suspicions regarding physical and/or substance abuse, and 10) concerns regarding suicide risk” [5].

According to New Zealand’s Health Information Privacy Code (HIPC), information found online through PTG constitutes health information if it is collected for the purpose of providing health services to the patient. This information does not necessarily have to be about the patient’s health to constitute ‘health information,’ so long as it is collected in the course of providing patient care. Searching for information about a patient for a reason unrelated to the provision of health care would be an unlawful breech of Rule 1 of the HIPC. If information is reasonably related to the provision of health services, then Rule 2(2)(f) allows for its collection from sources other than the patient or their representative so long as the information is ‘publicly available.’ According to Rule 3, the patient does not need to be informed when publicly available information is collected about them by means other than from the patient themselves or their representative [personal communication from Professor John Dawson to AC,12]. If information found through PTG is found to be untrue through litigation and lowered the patient’s reputation, then recording it in the patient’s notes may constitute defamation under New Zealand’s Defamation Act 1992. The healthcare professional could, however, quote whatever they found online [personal communication from Professor John Dawson to AC,13]. If PTG is conducted without consideration of these rules, there is a risk that medical practitioners and students may face legal ramifications, and therefore it is important to understand attitudes towards this behaviour and the reasons that might be given for engaging in PTG.

There are currently no data about the practice of PTG in a New Zealand context. Furthermore, though previous work has identified cases that demonstrate the need for guidance [4, 5], and data has been gathered about SNS use and attitudes towards PTG for routine matters [12], we are not aware of any previous work that has investigated student attitudes towards PTG, explored their reasons for considering it, and surveyed their SNS usage within the same cohort. We aimed to gain an understanding of (1) the extent of this practice in New Zealand, (2) the ethical and clinical issues that PTG presents, (3) the motivations and attitudes of the participants towards PTG, (4) and its association with online behaviours and SNS use, due to the role of SNSs as a source of online patient information and therefore its possible association with PTG. We investigated these aspects of PTG among trainee intern (final-year) medical students studying at New Zealand’s Otago Medical School, University of Otago.

## Method

### Participants

We sent an email invitation to 83 final year medical students at the Otago Medical School, University of Otago. This cohort was chosen for practical reasons; the students were largely centralised in Dunedin and we could contact them en masse. Also, they had several years of exposure to patients and hospital systems. This invitation included a hyperlink which directed respondents to an anonymous online survey. We personalised each hyperlink to allow for response rate tracking and reminder invitations. We sent a reminder email 1 week after the initial invitation and a final SMS text message reminder 2 days after this reminder email. Our survey was available over 2 weeks in January of 2016. The University of Otago Human Ethics Committee gave ethical approval for both the online survey and focus groups.

### Online survey

We collected and managed survey data using REDCap, hosted at the University of Otago [13]. REDCap is a secure, web-based application designed to support data capture for research studies. The survey consisted of 24 items, which included an electronic consent form. We collected basic demographic data, including age, gender, and ethnicity. We then explored the prevalence of PTG and attitudes towards the practice. We collected data on the prevalence and usage patterns of SNSs among the study cohort, due to a likely association with PTG. Several of these questions were based on a survey by Brisson et al., including a question around attitudes towards PTG and multiple questions around SNS usage patterns [12]. These questions provided a useful comparison between our results and external data. The SNS questions were chosen to reflect a broad understanding of the participants SNS use and online behaviours. The REDCap software automatically collated and analysed survey responses to provide us with descriptive statistics.

### Focus groups

We held two five-person focus group sessions in January of 2016. These focus groups were intended to explore the participants’ reasoning and motivations in greater depth than a survey could provide. Students indicated their willingness to participate in focus groups by responding to a question in the online survey and gave their contact email address for this purpose. All participants in the focus groups signed a paper consent form and completed a demographic questionnaire. Participants received edible incentives during each session. We recorded these sessions and used their subsequent transcriptions to conduct a thematic analysis according the approach detailed by Braun et al. [14]. To protect the participants’ identities during the focus groups, we asked them to use a randomly assigned number to identify themselves, in lieu of using their names. We prepared several thought provoking questions to stimulate conversation, though the subsequent discussion was allowed to take its own course. These questions investigated the risks and benefits of PTG, the likely effects of PTG, situations where it may be justified, what guidelines may be appropriate, and the ethicality of PTG. The first and second authors facilitated the focus groups. The second author was known to some participants in a teaching capacity. We used a qualitative descriptive approach to our analysis of the focus group discussions, focusing on the content of the data rather than the interpretive framework. The first and second authors independently coded our data by creating succinct codes, which identified ideas important to addressing our objectives, and applying them to recurring ideas throughout the transcript. We collated these codes and used them to identify broader patterns within the discussion.

## Results

### Online survey

Fifty-five of 83 eligible participants submitted a survey response. One participant declined to participate further after reviewing the electronic consent form, resulting in 54 valid survey responses and a response rate of 65.1%. Sixteen (19.3%) eligible participants were on overseas clinical placements during the study and may have had limited Internet access. Despite this potential barrier to completion for a number of respondents, a satisfactory response rate was achieved. Demographic data is presented in Table 1.

**Table 1:** Demographic characteristics of senior medical student survey respondents (*n* = 54), Dunedin, 2016

Ethnicity	n (%)	Gender	n (%)	Age	n (%)
New Zealand European	21 (38.9)	Female	29 (53.7)	20-23	21 (38.9)
Maori	2 (3.7)	Male	25 (46.3)	24-27	27 (50.0)
Asian	23 (42.6)	Other	0 (0.0)	28-31	2 (3.7)
Pacific Peoples	0 (0.0)			32-35	4 (7.4)
Other	8 (14.8)				

### Patient-targeted Googling

Nine (16.7%) respondents had conducted PTG; with only one respondent conducting PTG more than once per month. Thirty-four (75.6%) of those who had not conducted PTG indicated that they never would, with the remainder either stating they would or that they were undecided. Nine (16.7%) respondents indicated that they had seen someone else conduct PTG. Table 2 presents attitudinal responses to items relating to PTG. Respondents indicated their agreement with various statements on a five point Likert-type scale, from one (strongly agree) to five (strongly disagree). Table 2 indicates most (*n* = 36, 66.7%) respondents were concerned by PTG, while many (*n* = 25, 46.3%) respondents thought that they had received inadequate guidance on the issue.

**Table 2:** Senior medical student attitudes towards patient-targeted Googling (n = 54), Dunedin, 2016^a^

		n (%)	
	(1 or 2) agree or strongly agree	(3) undecided	(4 or 5) disagree or strongly disagree
I have no concerns about PTG.	10 (18.5)	8 (14.8)	36 (66.7)
I think PTG is appropriate for routine matters as opposed to a medical emergency.	0 (0.0)	7 (13.0)	47 (87.0)
I have received adequate guidance on PTG.	14 (25.9)	15 (27.8)	25 (46.3)
I would like the medical curriculum to provide more explicit guidelines on PTG.	29 (53.7)	17 (31.5)	8 (14.8)

*Abbreviations*: *PTG* Patient-targeted Googling

^a^The Likert-type scale has been collapsed to 3 points

### Social networking site usage

Table 3 shows the senior medical students had high levels of SNS usage, defined as the number of respondents who reported having at least one social media page (*n* = 51, 94.4%). Participants who were checking their SNSs more than five times per day were 1.83 times more likely to have conducted PTG than those using SNSs less frequently. Participants reported concerns about who they were connecting with online and the content of their SNS profiles. Twenty-one (38.9%) respondents’ SNS profiles contained information that they would not want a patient to see, and nine (16.7%) had information on their SNS profiles that they would not want a colleague to see. Twenty-eight (51.8%) had edited their SNS profiles due to potential career effects, though only three (5.6%) had deleted a profile for the same reason. Thirty-five (64.8%) respondents found it appropriate to accept a friend request from a superior, compared to 20 (37.0%) who considered it acceptable to send a friend request to a superior. Fifty (92.6%) found it inappropriate to accept a friend request from a patient, and only two (3.7%) had been approached by a patient online. Forty-seven (87.0%) respondents thought that doctors should not be able to send online friend requests to their patients.

**Table 3:** Social networking site use by senior medical students (n = 54), Dunedin, 2016

		n (%)
I have a social media page that contains personal information.	Facebook	49 (90.7)
	Google+	9 (16.7)
	Twitter	6 (11.1)
	personal blog	3 (5.6)
	none	3 (5.6)
	other	5 (9.3)
Frequency of access to personal social networking site pages.	never	2 (3.7)
	less than once per day	7 (13.0)
	more than once per day	19 (35.2)
	2-4 times per day	11 (20.4)
	more than 5 times per day	15 (27.8)
Awareness of the guidelines set out by the Code of Professional Conduct for Medical Students at the University of Otago which includes the use of social media and the Internet.		46 (85.2)

### Focus groups

Table 4 details several prominent themes, which emerged during the focus groups. Much of the conversation in the focus groups addressed attitudes towards PTG, but also reasons why students might consider this. Reasons identified included enhancing patient safety, but not pure curiosity, and seeking information to enhance student understanding of the situation leading to the patient’s presentation. Notably, most of the focus group participants had conducted PTG, in contrast to the low prevalence found in the online survey. It is difficult to say if this discrepancy is due to underreporting in the online survey, or a selection bias whereby participants who had conducted PTG were more interested in discussing it, nevertheless is suggests that estimates of the prevalence of PTG in the online survey data may be conservative. While participants did have reservations about PTG, they also commented on its positive potential in certain contexts, such as preventing harm to psychiatric patients or finding crucial information about unresponsive patients in an emergency department. Participants were concerned by the reliability of online information and with the ethics of PTG. They talked about potential issues with the ethical concepts of confidentiality and patient autonomy. Participants indicated that breaching these principles and the patient’s trust may result in damage to the doctor-patient relationship and consequently cause poorer patient health outcomes. Participants favoured more explicit teaching around PTG and had reservations about the level of guidance they had received within their training. The Otago Medical School curriculum does not specifically cover PTG; it does cover broader ethical teaching, the professional use of SNSs in healthcare, and professional boundaries. The intended use of information found online and the context of PTG were important to participants in deciding if the search was ethical and justified. In the reverse situation of PTG, participants held a negative view of patients searching for healthcare professionals online, as detailed by the ‘doctor-targeted Googling’ theme in Table 4. This is an interesting finding, though perhaps not unexpected. ‘Doctor-targeted Googling’ may not be consistent with the principles discussed in the wider literature around PTG, such as patient safety, as there is arguably little that it could contribute in this regard. Comments indicated that there may be some level of naivety among healthcare professionals and the general public regarding SNS privacy settings. Table 4 presents the themes identified in the focus groups, and representative attitudes encapsulated in each theme. The themes suggest that PTG is an issue of ethics and professionalism. They represent a cautious view of PTG among the participants, which is paralleled in the survey and in other studies [4, 9]. Furthermore, they identify some reasons why PTG might be considered appropriate, including if the intended use of the information is for patient safety, or where knowledge of patient identity may improve the safety of the healthcare team and other patients. Table 4. would appear to indicate that the ethicality and professionalism of PTG is heavily context dependent, though the motivation behind any search was consistently important to the participants in deciding if a search was justified.

**Table 4:** Salient themes derived through thematic analysis of focus group transcripts of ten senior medical students discussing social media and the Internet, Dunedin, 2016

Theme	Attitudes
Data reliability	Patient information found online was possibly inaccurate or misleading.
Education	While the participants had a good ethical foundation for considering PTG, they favoured more explicit teaching and guidelines. Teaching may be more practical than enforcing guidelines.
Relationships	PTG could damage the doctor-patient relationship, though may have some role in emotional closure after a patient’s death. Online information could have significant effects on the doctor’s perception of the patient and, therefore, on their health outcomes. Consented PTG may be beneficial to the doctor-patient relationship and to patient care.
Ethics	PTG may be ethical when conducted in the interest of benefiting the patient and preventing harm. PTG may breach patient confidentiality. It may also overstep the professional and personal boundaries of the doctor patient relationship.
Intended use	The intended use of online information is important. The healthcare professional should have a practical and ethical use in mind. Protecting patient safety is a use the participants felt was justified. Curiosity and voyeurism were treated with trepidation.
Guidelines	Participants wanted explicit guidelines, though recognised that these may be impractical to enforce.
Source	It would be more appropriate to ask the patient themselves or to ask an official organisation (e.g. Police) than to conduct PTG, in light of unreliable online information.
Doctor-targeted Googling	Participants viewed patients searching for their doctors online negatively, though they recognised this was a common practice, especially when patients chose a new healthcare providers or when they wanted to see public ratings of different doctors.
Role	Some comments indicated that it may not be the role of the healthcare professional to conduct PTG, and it may be better carried out by an official organisation such as the Police or Child Youth and Family.
Value	Most comments viewed PTG unfavourably, though some indicated that it would depend on the context.
Patient identity	This context of PTG may be different for famous patients due to more available information and prior knowledge of the person. Investigating a patient who has been in the justice system may be relevant to patient or staff safety. Reading online about a motor vehicle accident may provide a more in-depth case background. Conducting PTG in the interest of patient safety about psychiatric patients was viewed as reasonable.
Social media	Participants indicated that patients and healthcare professionals may be naive when it comes to keeping online information private.
Prevalence	Most focus group participants had conducted PTG.

*Abbreviations*: *PTG* Patient targeted Googling

## Discussion

PTG is an emerging topic of discussion, relevant to both the doctor-patient relationship and consequently to patient health outcomes. Our study offers data both qualitative and quantitative in nature, providing a comprehensive understanding of PTG in concert with SNS usage among senior New Zealand medical students. A recent increase in PTG rhetoric could be partly attributed to the permeation of Internet and SNS technologies into the health sector. We found this reflected in our study through high levels of SNS membership (*n* = 51, 94.4%) and the high frequency at which medical students check their SNS profiles (most check at least daily), this supports similar findings in other studies [1, 2].

Our finding that few students have conducted PTG is consistent with other data [9]. We believe this is reflective of generally unfavourable attitudes towards the practice, which are also seen in other studies [9, 12]. The Otago Medical School curriculum provides students with a useful ethical foundation for considering PTG, although most would like more explicit teaching on this issue. Personal ethical values are likely to affect a healthcare professional’s approach to PTG and may vary widely between individuals. Healthcare professional education could encourage a more homogenous approach to PTG and somewhat mitigate the effect of varying personal ethics. Healthcare professionals may be aided by guidelines or a pragmatic framework for considering PTG, such as the one detailed by Clinton et al. [4]. The framework offered by Clinton et al. addresses many of the issues and risks which arose in this study [4], though healthcare professionals using it should still pay heed to New Zealand’s privacy and defamation laws.

The veracity of information obtained through PTG is a pervasive concern in other studies and was commented on frequently in our focus groups [9, 15]. Healthcare professionals may find false or outdated information online; if a search was not disclosed to a patient, they would have no chance to corroborate or discredit the information found. Relying on potentially incorrect information to make assessments of risk could possibly lead to patients being significantly disadvantaged. Using nonspecific information to conduct a PTG search (e.g. the patient’s name) could result in finding the wrong person online, while using more accurate information obtained through the doctor-patient relationship (e.g. the patient’s home address) could be seen as abusing privileged information. Focus group participants discussed more reliable information sources than PTG, such as the Police or Child, Youth and Family (CYF, a New Zealand Government agency tasked with child protection). Participants indicated that these sources would be a more appropriate first approach than PTG. It was frequently assumed in our focus groups that consent is not usually obtained prior to conducting PTG; this could conflict with principles of autonomy and patient confidentiality.

PTG may present legal ramifications for healthcare professionals and their employers, particularly where untrue and derogatory information is repeated or recorded in the patient’s notes without clearly indicating the online source of the information [16]. New Zealand’s Health Information Privacy Code offers protection of the patient’s rights in this area [17]. A common justification of PTG in our focus groups and other studies is the issue of patient safety, especially when the patient is in a psychiatric care setting [4, 5, 15]. Motives involving curiosity and voyeurism are ethically dubious and cause for concern, they were treated with trepidation in our focus groups and in other research [4, 9]. PTG conducted for these motivations would be unlawful under the Health Information Privacy Code [17].

There is some evidence of an association between SNS use and likelihood of PTG, with high SNS users more likely to conduct PTG. Although this relationship is based on a small number of observations, due to the low prevalence of PTG identified by this sample, this is nonetheless an interesting observation. Perhaps one of the factors leading to PTG is simply a greater availability of the tools used to conduct it, and due to high SNS use in other parts of their lives there are fewer barriers to using these tools to gather patient information. The focus groups also revealed that despite having an awareness of guidelines, and a request for more guidance, there was also a recognition that guidelines on their own are insufficient to prevent inappropriate PTG. In addition to teaching students more about how to make ethically sound decisions, there may be a need to align ethics and professionalism more explicitly with teaching in digital literacies to ensure issues with PTG are minimized.

This study is perhaps limited by its relatively small sample size, which was selected with pragmatic intent. This study is, however, an important step in expanding the scope and extent of data available on the topic of PTG, and raises the possibility of larger studies in the future.

## Conclusions

Our analysis of the data from this study suggest that PTG may be viewed as ethically dubious in some situations, yet it may be crucial to patient care and safety in others. The decision to conduct PTG should be made in concert with careful consideration of ethical principles, motivations, and of the intended use of any information retrieved. Patients have an expectation of confidentiality and trust. This trust may be betrayed if healthcare professionals overstep the boundaries of the doctor-patient relationship by conducting PTG in a manner which does not align with the patient’s expectations, or with sound ethical and professional judgement. Healthcare professionals should pay close attention to the legality of their search with respect to the Health Information Privacy Code. Teaching or guidelines could mitigate the risk of legal issues and may be helpful to healthcare professionals facing ambiguous situations. PTG represents a risk of eroding the patient’s right to privacy and dignity, so should be approached with caution, in a robust and ethical manner. PTG should only be carried out to protect the wellbeing and safety of the patient, it should not be conducted out of curiosity or for reasons unrelated to the delivery of care. The association between PTG and SNS use raises the question of the extent to which online behaviours are responsible for PTG trends. This association warrants further investigation and could provide interesting future research. Investigating attitudes towards PTG among different healthcare professions could provide interesting data. Input from patients and the medico-legal sector may give a more extensive understanding of PTG.

## Abbreviations

CYF: Child, Youth and Family
HIPC: Health Information Privacy Code
PTG: Patient-targeted Googling
SNS: Social networking site
